# Social Dialogue and Psychosocial Risk Management: Added Value of Manager and Employee Representative Agreement in Risk Perception and Awareness

**DOI:** 10.3390/ijerph17103672

**Published:** 2020-05-22

**Authors:** Irene Houtman, Marianne van Zwieten, Stavroula Leka, Aditya Jain, Ernest de Vroome

**Affiliations:** 1 TNO, Work Health Technology, 2301 DA Leiden, The Netherlands; marianne.vanzwieten@tno.nl (M.v.Z.); ernest.devroome@tno.nl (E.d.V.); 2 Cork University Business School, University College Cork, T12 K8AF Cork, Ireland; stavroula.leka@ucc.ie; 3 Centre for Organizational Health and Development, School of Medicine, University of Nottingham, Nottingham NG8 1BB, UK; 4 Nottingham University Business School, University of Nottingham, Nottingham NG8 1BB, UK; Aditya.Jain@nottingham.ac.uk

**Keywords:** risk perception and awareness, psychosocial risk management, manager, employee representative, social dialogue, ESENER

## Abstract

The present study aimed to explore the added value of managers’ and employee representatives’ agreement in risk perception and awareness in explaining the management of more ‘subjective’ psychosocial risks as compared to the more ‘objective’ traditional OSH risks. The general assumption tested was whether the added value of agreement in risk perception and awareness between these parties would be larger for psychosocial risk management as compared to traditional OSH risk management. European Survey of Enterprises on New and Emerging Risks (ESENER-1) data were used from 7226 enterprises in which both managers and employee representatives were interviewed. Answers by employee representatives and managers to mirror questions on risk perception and awareness were used as independent variables, and answers to questions on risk management by the manager were used as dependent variables. Polynomial regression with response surface analysis was used. Differences in risk perception and awareness between managers and employee representatives explained more variance in psychosocial risk management as compared to more traditional OSH risk management. The implications of these findings and the importance of ‘social dialogue’ particularly in the case of psychosocial risk management as opposed to general OSH management are discussed.

## 1. Introduction

In recent decades, the need to manage risks associated with working conditions has become increasingly important, given the significant changes that have taken place in the world of work, including the nature of work and work organisations, contractual arrangements and new forms of employment, use of new technology and digitalisation and changes in workforce demographics [1]. Furthermore, during the last few decades there has been a shift away from traditional occupations (such as in industry) with high exposure to physical risks, towards other occupations (such as jobs in the service sector) with high exposure to psychosocial risks, which have contributed to decreases in accident rates but also to an increase in mental ill health [2,3,4].

Psychosocial risks arise from psychosocial hazards, which are aspects of work organisation, design and management that have the potential to cause harm to individual health and safety and organisational outcomes [1,5]. Psychosocial risks in the workplace are considered to be emerging and increasing [4,6,7,8,9,10]. Linked to psychosocial risks, issues such as work-related stress and workplace harassment and violence are now widely recognised as major challenges to occupational safety and health [1,5]. Psychosocial risks have been found to be significantly causally related to ill health including mental health disorders [11,12,13], cardiovascular disease [14,15] and musculoskeletal disorders [16,17]. They are also related to an increased risk of being absent as well as becoming disabled at a later stage [18,19].

A number of approaches have been developed and implemented by various stakeholders at the international, national, regional/sectoral and enterprise levels to manage psychosocial risks and promote better psychosocial work environments [20]. At the enterprise level, a substantial degree of diversity can be observed across such intervention strategies, with a common distinction being between organisational and individual orientations, or between primary-, secondary- and tertiary-level interventions.

While the need for effective interventions has been clear for some time, managing psychosocial risks effectively does not appear to be that simple, particularly as compared to traditional OSH risks [1,8]. One of the key reasons identified for this in the literature is that different stakeholders perceive these risks differently [21]. An important factor in these considerations is social dialogue and agreement in risk perception and awareness between employer and employee representatives [21,22,23], which may act as a driver as well as a barrier in psychosocial risk management. This study tested this assumption by exploring the added value of managers’ and employee representatives’ agreement in risk perception and awareness in explaining the management of psychosocial risks, which are considered ‘softer’ and more ‘subjective’, in comparison to that of traditional occupational safety and health (OSH) risks, which are considered ‘harder’ and more ‘objective’.

## 2. Stakeholder Perceptions and Psychosocial Risk Management

The anticipation, recognition, evaluation and control of risks arising in or from the workplace that could impair the safety, health and well-being of workers are the fundamental principles of the process governing occupational risk assessment and management (Plan, Do, Act, Check) [24,25]. The importance of hazard recognition and risk perception as drivers for the development and implementation of interventions to prevent and manage OSH risks is widely recognised [26]. It has been empirically shown that risk awareness is a major determinant of OSH risk management [27,28,29,30]. However, differences in risk perception between stakeholders (particularly employers/managers and employees/employee representatives) can have an impact on employer and employee commitment to engage in OSH risk management [31,32].

For instance, various studies have observed that managers and employees can develop different perceptions of the workplace safety climate, as managers are involved hands-on in work operations to a lesser extent than their employees, and therefore are not aware of possible short-cuts and rule violations occurring during operations. As a consequence, they might believe that safety climate is stronger than it really is and report higher safety climate scores than their employees [33,34,35]. Gittleman and colleagues [36] identified differences in perceptions of safety between employees and managers in construction, which were attributed to varying perspectives on performance and/or diverse opportunities to observe performance, while Marín et al. [37] found that managers reported higher safety climate scores than supervisors and construction workers, and discrepancies in perceptions of safety between workers and managers were positively correlated with company 3 year injury rate. The same might be true in relation to the perception of the psychosocial risks. A study that examined differences in perceptions of work environment factors between nurse managers and staff nurses showed that managers rated the psychosocial work environment more positively (i.e., low prevalence of psychosocial risks) than employees [38]. However, there is a knowledge gap relating to the possible differences in perceptions of psychosocial risks between managers and employees, which this study aimed to address.

Research also highlights the fact that differences in risk perceptions between stakeholders have a detrimental impact on the prioritisation of various OSH risks, and action taken to manage ‘new and emerging’ OSH risks as compared to ‘traditional’ OSH risks [1,8,21,39]. Houtman and colleagues [40,41] showed that in the case of psychosocial risk management, employee awareness adds to the explanation of risk management, whereas this was not the case in relation to physical risk awareness and management. Joint analyses of ESENER-2 (European Survey of Enterprises on New and Emerging Risks), the EWCS-2015 (European Working Conditions Survey) and the LFS 2013 ad hoc module (Labour Force Survey) showed that the presence of mental health complaints additionally explained psychosocial risk management, whereas physical health complaints did not additionally explain general OSH and musculoskeletal disorder (MSD) risk management [28]. This suggests that employers and their representatives require more convincing in order to engage in psychosocial risk management as compared to OSH or MSD risk management.

In addition, the main barriers to the development and implementation of interventions for psychosocial risk management include a lack of stakeholder commitment, perceived sensitivity of psychosocial issues and the perception of stakeholders that interventions for managing psychosocial risks are expensive, complex and require high-level expertise [9,27]. Despite the fact that these types of intervention may not necessarily be expensive, studies focusing on social dialogue at European, national and enterprise levels have highlighted that awareness in relation to psychosocial risks varies greatly among EU member states as well as among stakeholders, and suggest that agreement is one of the key catalysts for action at all these levels [21,22,23,42].

According to the International Labour Organisation (ILO), social dialogue refers to all types of negotiation, consultation or exchange of information between or among governments, employers and workers, whether bipartite or tripartite, and informal or institutionalised. Social dialogue can take place at the national, regional or enterprise level, and bipartite social dialogue most commonly deals with working conditions and terms of employment as well as the relationships between workers and employers [43]. The importance of social dialogue in the OSH arena and its positive impact on working conditions in Europe is recognised by all stakeholders [44]. To address the challenge posed by psychosocial risks in the EU, the European cross-sectoral social partners have signed autonomous agreements, the Framework Agreement on Work-related Stress [45] and the Framework Agreement on Violence and Harassment at Work [46]. These agreements, which are implemented not by legislation at EU level, but rather by national social partner organisations in accordance with national procedures and practices, represent a commitment to the development and application of their content by social partners at the national, sectoral and company level [3].

Studies by both Eurofound and EU-OSHA have provided evidence that, in order to be effective and to see real improvements in working conditions in general, different actors have to work together in their joint interests and to achieve a shared understanding of challenges and expectations of a win-win situation, beneficial for both sides. In this regard, evidence has been found for the contribution of social dialogue at sectoral and company level to the improvement of working conditions [47]. Furthermore, ESENER data show that management commitment and worker participation are important for effectively managing psychosocial risks at work [9,27].

The evidence also suggests that psychosocial risk management may particularly benefit from more social dialogue through employee participation and employer commitment, starting with risk awareness. This may be especially important since more traditional OSH risks are considered more ‘objective’, whereas psychosocial risks, by nature, might entail a greater level of ‘subjectivity’. Jespersen and colleagues even argued that psychosocial risks share important features of ‘wicked problems’, since they are often multi-causal, contextualised, rarely directly visible (often not directly observable) and highly political or politicised (relating, for instance, to the employer prerogative) [48]. Thus, this study addressed the need for more concrete and quantitative evidence to show that worker–manager agreement is an important driver for risk management, particularly for psychosocial risk management. This is important because despite the recognised potential negative impact of psychosocial risks on employee health, safety and well-being and organisational performance, action taken to manage these risks has not improved adequately at the international [1], European [49] or national levels [39].

Limited data allow testing of the level of agreement between stakeholders on traditional OSH risks in comparison to psychosocial risks and its impact on actions taken. In this paper, we tested this by analysing data from the first European Survey of Enterprises on New and Emerging Risks (ESENER-1), in which managers and employee representatives were asked both about risk perception and awareness and risk management. By using polynomial regression with response surface analysis, we tested the explanatory power of agreement in risk perception and awareness between managers and employee representatives from the same enterprise in risk management of more traditional OSH risks as compared to psychosocial risks.

It was hypothesised that (1) the discrepancy in risk perception and awareness between managers and employee representatives would be higher when it came to psychosocial risks, as compared to general OSH risks.

It was further hypothesised that (2) the pattern of the relationship between risk perception and awareness of both traditional OSH risks and of psychosocial risks for managers and employee representatives and risk management measures taken would be such that (a) agreement in perception and awareness would be positively related to measures taken (Hypothesis 2a), (b) the degree of discrepancy or disagreement on risk perception and awareness would be negatively related to measures taken (Hypothesis 2b), and (c) a higher awareness on the part of the manager (as compared to the employee representative in the same enterprise) would be more positively related to measures taken than a higher awareness on the part of the employee representative (as compared to the manager in the same enterprise; Hypothesis 2c).

Finally, it was hypothesised that (3) the degree of agreement or discrepancy between managers and employee representatives would be more strongly associated with risk management in the case of psychosocial risks as compared to general OSH risks (Hypothesis 3).

## 3. Materials and Methods

### 3.1. Design

Data for this study were derived from the first European Survey of Enterprises on New and Emerging Risks (ESENER-1) conducted by the European Agency for Safety and Health at Work [7]. The ESENER explores the views of managers and employee representatives on how health and safety risks are managed in their workplace, with a particular focus on psychosocial risks. The survey was conducted in 2009 on a representative sample of organisations and enterprises with ten or more employees in each of the (then) 27 EU Member States, as well as in Croatia, Turkey, Norway and Switzerland. It covered both private and public organisations from all sectors of activity except for agriculture, forestry and fishing, private households and extraterritorial organisations. Data were collected through computer-assisted telephone interviewing (CATI). In each establishment, one management interview was conducted. The respondent for the management interview was defined as the most senior manager coordinating safety and health activities in the establishment. In all organisations where a formal health and safety employee representative existed at the local level, one additional interview with the health and safety representative was conducted [50]. The respondent for this interview was an employee representative, i.e., not a health and safety specialist determined by the management. As a general rule, the respondent of first choice for the employee representative interview was the spokesperson of the employee side within the health and safety committee. Health and safety committees (formal or informal) are established in several enterprises across countries in Europe, and are working groups that deal with all issues of safety and health concern in an establishment.

### 3.2. Study Population

A total of 28,649 managers and 7226 employee health and safety representatives were interviewed in ESENER-1. The selection of organisations for this paper was first restricted to those for which both the management interview and the interview with the employee representative were present, but after list-wise deletion in order to deal with missing data, analyses were ultimately performed on a sample of 7068 organisations.

A comparison of the selected organisations with the remaining group of organisations showed that the selected organisations were significantly larger (more often more than 50 employees) and older (established before 1990). The selected organisations more often came from public administration, health, social work and other community, social and personal service activities, manufacturing, or electricity, gas and water supply, and came less often from construction, wholesale and retail trade, repair of motor vehicles, motorcycles and personal and household goods, hotels and restaurants and real estate, renting and business activities. Furthermore, the selected organisations more often came from Denmark, Germany, Finland, Ireland, Italy, Sweden, Bulgaria and Estonia.

The response rate varied per country (see Reference [7], p. 95) and ranged from 14% in Luxembourg and Croatia to 59% in Greece. Table 1 presents the sample characteristics. EU-OSHA stated that the large discrepancies in response rates were mainly due to national differences in the willingness amongst enterprises to participate in telephone interviews. In order to reproduce real quantitative proportions for cross-national analysis, an ‘international weighting’ was used to adjust the national sample sizes. In the case of this study, the weighting accommodated an establishment proportional analysis.

### 3.3. Psychosocial Risk Perception and Awareness

The independent variables were the answers to the mirror questions in the manager and employee representative questionnaires on risk perception and awareness. Managers as well as employee representatives were asked whether any of the following psychosocial risks were a (major, some or no concern, or ‘not applicable’) concern in their enterprise: time pressure; poor communication between management and employees; poor cooperation amongst colleagues; lack of employee control in organising work; job insecurity; having to deal with difficult customers, patients, pupils etc.; problems in supervisor–employee relationships; long or irregular working hours; an unclear human resources policy; and discrimination. Each person’s response to the questions on risk perception and awareness were averaged to arrive at an individual scale score. A scale score was only calculated if the person answered at least seven out of ten questions on psychosocial risks. Cronbach’s alphas were 0.72 for managers and 0.75 for employee representatives.

### 3.4. General Occupational Safety and Health Risk Perception and Awareness

Managers and employee representatives were asked whether the following issues were a (major, some or no concern, or ‘not applicable’) concern in their enterprise: dangerous substances; accidents; noise and vibration. Each person’s response to the questions on risk perception and awareness were averaged to arrive at an individual scale score. A scale score was only calculated if the person answered at least two out of three questions. Cronbach’s alphas were 0.68 for managers and 0.70 for employee representatives.

### 3.5. Psychosocial Risk Management

Managers were asked whether in the last three years their enterprise had used any of the following measures to deal with psychosocial risks: changes to the way work is organised, redesign of the work area, confidential counselling for employees, set-up of a conflict resolution procedure, changes to working time arrangements, and provision of training (yes, no or not applicable). We averaged each person’s responses across items to arrive at a scale score for each person. A scale score was calculated only if the person answered at least four out of six questions (Cronbach’s alpha = 0.71).

### 3.6. General Measures for Traditional OSH Management

Managers were asked whether workplaces in their establishment are regularly checked for safety and health as part of a risk assessment or similar measure (yes, no or not applicable). They were also asked whether equipment and working environment are routinely considered in these checks and whether actions have been taken as follow-up to these checks (yes, no or not applicable). Using these three variables, we calculated a new variable with a dichotomous outcome: no, no regular check for safety and health and, yes, a regular check including equipment and working environment and follow-up actions.

### 3.7. Data Analysis

We aimed to explore how agreement or discrepancies in risk perception and awareness between managers and employee representatives were associated with risk management. In order to do this, a stepwise approach was followed. First, we determined a discrepancy score between risk perception and awareness of the manager and employee representative from the same organisation, both on psychosocial risk perception and awareness and general OSH perception and awareness. A discrepancy score was determined for each organisation, based on the mean and standard deviation of each organisation. We first standardised the scores for each predictor variable [51]. A discrepancy was significant if a standardised score on one predictor variable (risk perception and awareness by the manager) was half a standard deviation or more above or below the standardised score on the other predictor variable (risk perception and awareness by the employee representative).

Secondly, before conducting the regression analysis, the predictors were centred on the scale midpoint [52]. This is essential for the proper interpretation of regression results and reduces the potential for multicollinearity [53]. Thirdly, we conducted a polynomial regression analysis and response surface analysis [52,54,55]. This procedure offers several advantages over the use of the difference scores: ‘difference between manager and employee representative’ [56]. Using polynomial regression and subsequent response surface analysis, one can examine how agreement and disagreement between two predictor variables relate to an outcome. One can also examine how the direction of discrepancy between two predictor variables relates to an outcome.

As for the polynomial regression with response surface analysis, the independent variables were the two centred predictor variables (the perception and awareness of the manager and the perception and awareness of the employee representative), the product of the centred predictor variables, the square of one predictor variable, and finally the square of the other predictor variable. The dependent variable was ‘taking measures to manage (either psychosocial or traditional OSH) risks’. If the variance in the outcome variable explained by the regression equation (R^2^) was significantly different from zero, the results of the polynomial regression were evaluated regarding four so-called ‘surface test values’ (Edwards, 2002). These four values (a1, a2, a3, and a4) basically reparametrise the beta coefficients of the regression equation Z = b0 + b1 × X + b2 × Y + b3 × X^2^ + b4 × XY + b5 × Y^2^ + e. The values were used for response surface analysis, which graphs the results of polynomial regression analyses in a three-dimensional space in order to facilitate their interpretation. This provides a nuanced view of the relationship between a combination of the predictor variables and the outcome variable.

## 4. Results

The means, standard deviations and univariate relations among the variables used in this study are shown in Table 2. Psychosocial risk perception and awareness in general was lower than OSH risk perception and awareness. However, overall, employee representatives were somewhat more aware of psychosocial risks, whereas the opposite was reported in the case of general OSH risks, where managers reported a higher risk perception in their establishment. In addition, more measures directed at general OSH management were taken as compared to psychosocial risk management.

Furthermore, Table 2 shows that for both managers and employee representatives, significant positive bivariate relationships were found between psychosocial risk perception and awareness and psychosocial risk measures. Significant positive bivariate relationships also existed between general OSH risk perception and awareness, of both managers and employee representatives, and ‘general OSH measures’. However, psychosocial risk perception and awareness was more strongly correlated with psychosocial risk measures than is traditional OSH risk perception and awareness of ‘general OSH measures’.

### 4.1. Degree of Agreement and Discrepancies in Risk Perception and Awareness

The degree of agreement and disagreement or discrepancies in risk perception and awareness is shown in Table 3. It was found that in about one-third (36%) of the organisations, the managers and employee representatives agreed on the presence of psychosocial risks in their organisation, whereas this was almost 50% in the case of more traditional OSH risks. Regardless of the type of risk, the disagreement between managers and employee representatives was evenly distributed across the sample (with about the same number of organisations in which the manager reported higher risk perception and awareness and those in which the employee representative did so).

For both psychosocial risk and traditional OSH risk perception and awareness, it was apparent that employee representatives perceive, and are aware of, the risk almost as often as managers.

### 4.2. Psychosocial Risk Perception and Awareness Explaining Psychosocial Risk Management

**Figure 1:**
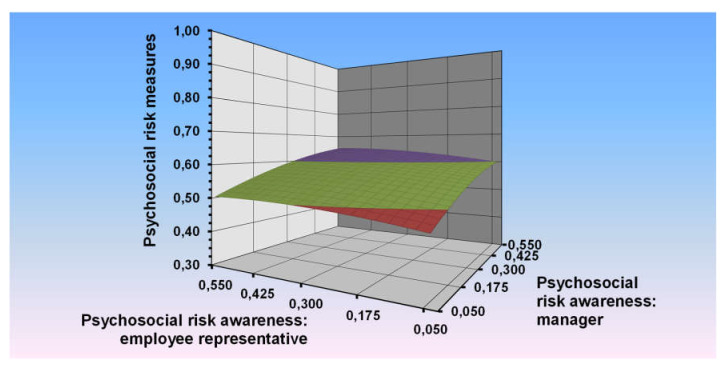
Psychosocial risk measures explained by the combination of the psychosocial risk perception and awareness of the manager and the employee representative.

By means of polynomial regression procedures with response surface testing, we first examined how both the psychosocial risk perception and awareness of the manager and the employee representative act and interact together as explanatory variables of psychosocial risk management measures taken in the last three years. Table 4 summarises the results of this analysis. The results are shown graphically in Figure 1.

Values of a1 and a2 were examined to evaluate how agreement in perception and awareness between managers and employee representatives, respectively, related to the outcome. Value a1 defines the slope of the line of perfect agreement, a2 defines the curvature along the line of perfect agreement. If a1 differs significantly from zero and a2 does not, there is a linear slope along the line of perfect agreement. A significant negative value for a2 indicates a concave surface along the line of perfect agreement, while a significant positive value of a2 indicates a convex surface.

A significant positive value for a1 (0.37, *p* < 0.001) and a significant negative value for a2 (−0.47, *p* < 0.001) indicated a concave surface (downward curving) along the line of perfect agreement. For organisations in which the psychosocial risk perception and awareness of the manager and the employee representative were in agreement, more psychosocial risk management measures were taken.

Figure 1 also provides information on the effect of discrepancy (degree and direction) between the psychosocial risk perception and awareness of the manager and the employee representative on psychosocial risk management measures. The effect of discrepancy was inspected on the line of disagreement (perception and awareness manager = −perception and awareness employee representative). The linear slope of the line is indicated by a3, while the curvature of this line is indicated by a4. If a3 differs significantly from zero and a4 does not, there is a linear slope along the line of disagreement. The significant positive a3 value indicated that more psychosocial risk management measures were taken when the direction of the discrepancy was such that the perception and awareness of the manager was higher than the perception and awareness of the employee representative than vice versa. Additionally, a curve along the line of incongruence is indicated by a4, where a negative value indicates a concave surface along this line and a positive value indicates a convex surface. It appears that the a4 value was negative and significant, indicating that as the discrepancy between the psychosocial risk perception and awareness of the manager and the employee representative increases, psychosocial risk management measures taken decrease.

### 4.3. Perception and Awareness of Traditional OSH Risks and Traditional Risk Management

**Figure 2:**
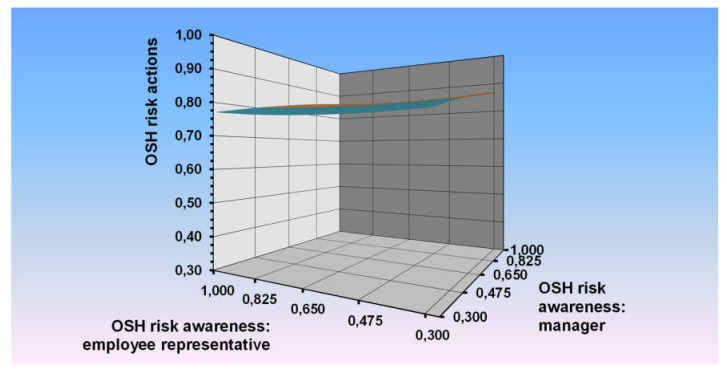
OSH risk actions explained by the combination of the OSH risk perception and awareness of the manager and the employee representative.

By means of polynomial regression procedures with response surface testing, we also examined how both general OSH risk perception and awareness of the manager and the employee representative act together and interact as explanatory variables of traditional risk management. Table 5 summarises the results of this analysis. Results are shown graphically in Figure 2.

First of all, it is apparent that the explained variance for general OSH management was much less than the explained variance for psychosocial risk management (2% versus 9%). To evaluate how agreement in perception and awareness between managers and employee representatives respectively related to the outcome, values a1 and a2 were examined. As before, value a1 defines the slope of the line of perfect agreement, a2 defines the curvature along the line of perfect agreement. The significant positive value for a1 indicated a linear slope along the line of perfect agreement. For enterprises in which OSH perception and awareness of the manager and the employee representative agreed, follow-up actions in the area of general traditional OSH management were reported to be taken more often.

The significant positive a3 value indicated that general OSH measures were more often taken when the direction of the discrepancy was such that the perception and awareness of the manager was higher than the perception and awareness of the employee representative than vice versa. The surface value a4 was not significant, which means that this relation was linear.

Overall, the first hypothesis was confirmed, indicating that risk perception agreement and awareness between managers and employee representatives was found to be higher for general OSH risks (49%) as compared to psychosocial risks (36%). In relation to the second hypothesis, results showed that for both OSH and psychosocial risk management, agreement in perception and awareness between managers and employee representatives was found to be positively related to measures taken, although the impact was considerably larger for psychosocial risks (confirming Hypothesis 2a). Furthermore, a discrepancy or disagreement on risk perception and awareness between managers and employee representatives was negatively related to taking measures (confirming Hypothesis 2b). In addition, the risk perception and awareness of the manager appeared to be more decisive in stimulating risk management as opposed to that of the employee representative (confirming Hypothesis 2c). Overall, analyses showed that all these relationships were stronger for psychosocial risk management as compared to OSH risk management (confirming Hypothesis 3).

## 5. Discussion

The aim of this study was to explore empirically whether the degree of psychosocial risk perception and awareness between managers and employee representatives within the same organisations is an important explanatory variable in risk management, but differs according to the type of OSH risk. Agreement in risk perception and awareness between managers and employee representatives was hypothesised to be more important in relation to ‘softer’ psychosocial risks as compared to the more traditional OSH risks, like physical and ambient risks.

First of all, and in accordance with our hypothesis, there was less agreement between managers and employee representatives on the presence of psychosocial risks in the organisation as compared to more traditional OSH risks. This may be related to the level of awareness, linked to training of both managers and employees [1]. It may also reflect the fact that employees might have better understanding of psychosocial risks involved in their work, while this may be less visible to managers (as other types of OSH risks might be), as also highlighted in previous research on occupational safety risks [33]. Managers may also underestimate psychosocial risks in comparison to other OSH risks [8,39], or they may perceive them to be more sensitive and relevant to the individual and not the organisation [1,7]. However, in case of a discrepancy in perception and awareness, situations in which the manager perceived the risk to be present but the employee representative did not were almost as prevalent as situations in which the employee representative perceived the risk to be present and the manager did not. Rather unexpectedly, this response pattern was about the same for perception and awareness of psychosocial risks as for the perception of more traditional OSH risks. Again, managers might not have a good understanding of the situation ‘on the ground’ and real issues workers are dealing with. On the other hand, managers might sometimes have a more strategic perspective and sight of emerging issues that can present challenges [57].

By means of polynomial regression procedures with response surface testing, perception and awareness of psychosocial risks of managers and employee representatives were jointly taken into consideration as explanatory variables of risk management, as related to both psychosocial risk management and to more traditional OSH risk management. The analyses presented in this paper confirmed that risk perception and awareness is a major explanatory variable of risk management. It indeed appeared to be a more important predictor for psychosocial risk management as compared to more traditional OSH risk management, and the added value of agreement between managers and employee representatives was particularly important for psychosocial risk management. This might again relate to the nature of psychosocial risks, which are often considered more sensitive issues and are less visible in organisations [1,7]. Therefore, when both managers and employee representatives perceive them to be important, the likelihood of taking action to address them increases, thereby supporting the importance of social dialogue at the enterprise level for psychosocial risk management, as highlighted in previous research [22].

Factors that add to the impact of social dialogue, which have been shown to be important in the effective implementation of psychosocial risk management and which are identified as ‘active ingredients’ for good psychosocial risk management are employer commitment as well as employee commitment and participation in decision-making in relation to the psychosocial risks present in the establishment, and open and transparent communication [9]. A better mutual understanding of what constitutes the difference in risk perception by workers or worker groups as well as management may make it possible to design comprehensive risk-management interventions that involve managers, supervisors and workers. This was recently illustrated by a study which showed that individual group perceptions of safety climates were not related to the injury rate (of construction companies), whereas worker–manager discrepancies in perception of safety risk were. The authors concluded that discrepancies between workers’ and managers’ perspectives with respect to risk management priorities may be important regarding effective mitigation of these risks. Each group provided a unique perspective with respect to risk management priorities [37].

The importance of social dialogue for the quality of risk management in general and psychosocial risk management, in particular, has been highlighted before [21,22]. This was also supported in the current study with a focus on the enterprise level. In general, the risk perception and awareness of the manager was highly important for both traditional OSH risk management and psychosocial risk management. However, the risk perception and awareness of employee representatives significantly added to the explanation of psychosocial risk management, both as a main effect and in interaction with the manager perception, but did not add to the explanation of traditional OSH risk management. These findings suggest that social dialogue between managers and employee representatives at the enterprise level is potentially a strong vehicle to discuss and raise awareness particularly of psychosocial risks, with a view to promoting psychosocial risk management. This is crucial given the diversity in awareness and prioritisation of psychosocial risks that exist across EU member states [1,21]. Findings from additional past research repeatedly show that involvement of employees next to employer commitment and participation at enterprise level are important factors in realising effective psychosocial risk management [28,57,58].

The strength of this study was that the database used is quite large and originally more representative at the establishment (management representative) level, as well as unique in that it includes information from both managers and employee representatives on the same issues of risk perception and risk management. However, because we wanted to use information from both managers and employee representatives, our sample ended up being restricted to those enterprises where both interviews were available through the ESENER database. These enterprises tended to be older and larger. Furthermore, ESENER-1 did not include micro-enterprises which constitute the large majority of enterprises in Europe and have been shown to less often take measures to manage OSH risks, including psychosocial risks [7,8,10].

While the ESENER-1 survey was conducted in 2009 during the economic recession that constituted a massive shock to most firms all over the Western world, we do not believe this had an impact on the study findings. During the recession, OSH management, including psychosocial risk management, was found to be deteriorated [59]. During the same time, no new legislation was introduced on psychosocial risk management at the EU level. However, there was more prioritisation of psychosocial risks due to national responses to the two Framework Agreements on Work-Related Stress and Violence and Harassment at Work [60,61,62,63], through the Joint Action on Mental Health [64], and as an outcome of the evaluation of the EU OSH strategy [65], which highlighted the importance of the need for more focus to be placed on managing psychosocial risks [66].

Based on previous research on similar combined datasets, we expect that selection bias will impact the reported prevalence of (psychosocial) risk awareness and management activities taken in the enterprise; however, its impact on the correlation and regression results between risk awareness and risk management is expected to be much less [40]. In addition, the fact that the survey asked managers if any measures had been taken in the last three years in their enterprise may have resulted in recall bias due to the fact that risks or management activities may have been not or be falsely remembered. In addition, assessing the perceptions of single key informants from both management and employee representatives may have resulted in bias as well, since these respondents may not have been well informed or may just not have been aware of risks and/or of measures taken. However, the impact of both recall bias and single key informants would have increased random error, and thus may have reduced the likelihood of finding effects. Random error will generally not result in finding effects that are not present in the population. This supports the fact that the findings presented here are robust findings of real associations in a somewhat selective but large sample of European establishments.

Another weakness of this study was the fact that most of the general OSH measures that could be considered in this study are mandatory by law. This means that they are less debatable than measures directed at psychosocial risks, which depend on the situation in the particular organisational context. However, even general OSH measures that are mandatory by law still appear not to be practised in all organisations [7]. In addition, since this study was cross-sectional in nature, we should be cautious not to interpret the relations studied as causal. Although it is tempting to think that perception and awareness lead to (planned) action, it still may also be the case that the measures themselves (e.g., in the case of general OSH management thus included a risk assessment) result in (general or specific) OSH awareness. The latter relationship may be less probable in the case of psychosocial risk management, as these measures were operationalised in the present study as, for example, work redesign, or training, which can much more likely be considered consequences of risk perception and awareness.

## 6. Conclusions

For future research, the above findings suggest comparing the added value of risk perception and awareness of employees as well as employers and their representatives regarding both psychosocial and other types of OSH risk management, preferably in a prospective design. It may additionally be important for reasons of impact of the results to include context variables that are relevant to the present and future determinants of the labour market. For example, the degree to which these relationships are causal and dependent on economic growth, as well as on employee characteristics, such as age, educational level and health status, could be examined.

These findings indicate that perception and awareness of risks significantly explains risk management, particularly in the case of psychosocial risk management. Furthermore, agreement in perception and awareness between managers and employee representatives is of additional importance for psychosocial risk management. The present analyses support the notion that social dialogue at the enterprise level is much more essential for ‘soft’ psychosocial risks than for more traditional OSH risks. It is recommended that risk perception and awareness are addressed as a priority issue through social dialogue initiatives to promote psychosocial risk management at European, national and enterprise levels.

## Figures and Tables

**Table 1:** Sample characteristics.

Enterprise Characteristics		Interview with Both Management and Employee Representative (%)	Only Management Interview (%)	Total (%)
**Company size**	10–19	13.7 ▼	29.6 ▲	25.6
	20–49	22.3 ▼	29.1 ▲	27.4
	50–99	16.3 ▲	14.6 ▼	15.1
	100+	47.7 ▲	26.7 ▼	32.0
**Establishment founded ^1^**	Before 1990	61.6 ▲	51.5 ▼	53.6
	Between 1990 and 2005	33.9 ▼	43.3 ▲	41.1
	After 2005	3.9 ▼	4.6 ▲	4.5
**Does this establishment belong to the public sector? ^1^**	Yes	26.3 ▲	20.1 ▼	21.7
	No	73.3 ▼	79.5 ▲	77.9
Sector (NACE)				
C—Mining and quarrying		0.6	0.7	0.7
D—Manufacturing		32.7 ▲	28.6 ▼	29.6
E—Electricity, gas and water supply		2.0 ▲	1.0 ▼	1.2
F—Construction		8.0 ▼	10.2 ▲	9.6
G—Wholesale and retail trade; repair of motor vehicles, motorcycles and personal and household goods		11.1 ▼	15.9 ▲	14.7
H—Hotels and restaurants		1.9 ▼	3.8 ▲	3.3
I—Transport, storage & communication		4.6	4.6	4.6
J—Financial intermediation		2.4	2.5	2.5
K—Real estate, renting & business act.		8.5 ▼	9.8 ▲	9.5
L—Public administration and defence; compulsory social security		6.1 ▲	5.0 ▼	5.3
M—Education		7.9	7.4	7.6
N—Health and social work		9.8 ▲	6.7 ▼	7.5
O—Other community, social and personal service activities		4.6 ▲	3.8 ▼	4.0
**Country**				
BE		3.2 ▼	3.9 ▲	3.7
DK		7.2 ▲	2.3 ▼	3.5
DE		6.9 ▲	4.7 ▼	5.3
EL		1.8 ▼	4.1 ▲	3.5
ES		5.2	5.6	5.5
FI		9.5 ▲	1.5 ▼	3.5
FR		5.4	5.2	5.2
IE		2.3 ▲	1.6 ▼	1.8
IT		6.7 ▲	4.8 ▼	5.2
LU		1.5	1.8	1.7
NL		2.9 ▼	3.7 ▲	3.5
AT		2.3 ▼	4.0 ▲	3.6
PT		0.7 ▼	4.5 ▲	3.5
SE		7.2 ▲	2.2 ▼	3.5
UK		4.2 ▼	5.6 ▲	5.2
BG		3.2 ▲	1.3 ▼	1.7
CY		0.7 ▼	2.1 ▲	1.8
CZ		2.5 ▼	3.9 ▲	3.5
EE		2.7 ▲	1.4 ▼	1.7
HU		2.9 ▼	3.8 ▲	3.6
LV		1.7	1.8	1.8
LT		1.1 ▼	2.0 ▲	1.8
MT		0.8 ▼	1.3 ▲	1.2
PL		5.0	5.3	5.2
RO		1.8	1.8	1.8
SK		0.9 ▼	2.1 ▲	1.8
SI		1.1▼	2.1 ▲	1.8
TR		1.8 ▼	6.4 ▲	5.2
HR		1.7	1.7	1.7
CH		1.8 ▼	4.2 ▲	3.6
NO		3.4	3.3	3.3

Note: ▼ and ▲ indicate a significantly lower/higher percentage as compared to the total average, determined using chi-square tests. ^1^ Not all percentages add up to 100% as not all respondents answered this question/did not know.

**Table 2:** Means, standard deviation and intercorrelations among study variables (*n* = 6882).

	Variable	*M*	*SD*	1	2	3	4	5
1.	Psychosocial risk perception and awareness, employee representative	0.34	0.25	-				
2.	Psychosocial risk perception and awareness, manager	0.31	0.23	0.21 **	-			
3.	Occupational safety and health perception and awareness, employee representative	0.70	0.36	0.16 **	0.08 **	-		
4.	Occupational safety and health perception and awareness, manager	0.72	0.35	0.07 **	0.18 **	0.43 **	-	
5.	Psychosocial risk measures, manager	0.47	0.31	0.15 **	0.27 **	0.06 **	0.09 **	-
6.	Occupational safety and health measures, manager	0.61	0.35	0.03 **	0.00	0.06 **	0.09 **	0.17 **

Note: ** *p* < 0.01

**Table 3:** Agreement in psychosocial risk perception and awareness and OSH risk perception and awareness between managers and employee representatives.

Agreement Groups	Psychosocial Risk Perception & Awareness (%)	OSH Risk Perception & Awareness (%)
Manager perception and awareness and employee representative perception and awareness in agreement	36	49
Employee representative perception and awareness higher than manager perception and awareness	32	24
Manager perception and awareness higher than employee representative perception and awareness	32	27

Note: agreement was defined when a standardised score on one predictor variable (as perceived by the manager) was less than half a standard deviation above or below the standardised score on the other predictor variable (as perceived by the employee representative).

**Table 4:** Response surface tests for psychosocial risk perception and awareness of the manager and the employee representative.

	Psychosocial Risk Measures
Variable	b (SE)
1. Perception & awareness manager	0.28 (0.02) ***
2. Perception & awareness employee representative	0.09 (0.02) ***
3. Perception & awareness manager squared	−0.32 (0.05) ***
4. Perception & awareness manager × Perception & awareness employee representative	0.04 (0.06)
5. Perception & awareness employee representative squared	−0.20 (0.05) ***
R^2^ (explained variance)	9.0%
Surface tests	
a1 (the linear slope of the line of perfect agreement)	0.37 ***
a2 (the curvature of the line of perfect agreement)	−0.48 ***
a3 (the linear slope of the line of disagreement)	0.19 ***
a4 (the curvature of the line of disagreement)	−0.56 ***

Note: b = unstandardised regression coefficient. a1 = b1 + b2 where b1 is the regression coefficient for the perception and awareness of the manager and b2 is the regression coefficient for the perception and awareness of the employee representative. a2 = b3 + b4 + b5, where b3 is the regression coefficient for perception and awareness of the manager squared, b4 is the regression coefficient for the cross product of perception and awareness of manager and employee representative, and b5 is the regression coefficient for perception and awareness of the employee representative squared. a3 = b1 − b2. a4 = b3 − b4 + b5. *** *p* < 0.001.

**Table 5:** Response surface tests for OSH perception and awareness of the manager and the employee representative.

	‘Perceived Impact of General Occupational Safety and Health Policy’
Variable	b (SE)
1. Perception & awareness manager	0.15 (0.02) ***
2. Perception & awareness employee representative	0.01 (0.02)
3. Perception and awareness manager squared	−0.07 (0.05)
4. Perception & awareness manager × Perception & awareness employee representative	−0.05 (0.04)
5. Perception & awareness employee representative squared	0.10 (0.05) *
R^2^ (explained variance)	2.0%
Surface tests	
a1 (the linear slope of the line of perfect agreement)	0.16 ***
a2 (the curvature of the line of perfect agreement)	−0.02
a3 (the linear slope of the line of disagreement)	0.14 ***
a4 (the curvature of the line of disagreement)	0.09

Note: b = unstandardised regression coefficient. a1 = b1 + b2 where b1 is the regression coefficient for the perception and awareness of the manager and b2 is the regression coefficient for the perception and awareness of the employee representative. a2 = b3 + b4 + b5, where b3 is the regression coefficient for perception and awareness manager squared, b4 is the regression coefficient for the cross product of perception and awareness of manager and employee representative, and b5 is the regression coefficient for perception and awareness of the employee representative squared. a3 = b1 − b2. a4 = b3 − b4 + b5. * *p* < 0.05, *** *p* < 0.001.
